# Facile synthesis of functionalized polyglycidyl methacrylate-magnetic nanocomposites for enhanced uranium sorption†

**DOI:** 10.1039/c9ra06874k

**Published:** 2019-11-27

**Authors:** Ahmed A. Galhoum

**Affiliations:** Nuclear Materials Authority P. O. Box 530, El-Maadi Cairo Egypt galhoum_nma@yahoo.com

## Abstract

Designing and fabricating nanocomposite magnetic sorbents (with more accessible active sites for achieving high sorption capacities, selectivity and rapid kinetics) has become an impending challenge in the removal of radionuclides. Two core–shell multifunctional magnetic-nanocomposites have been prepared suitably to be used as sorbents using facile two-step processes. In the first step, after synthesis of parent PGMA microparticles (by a dispersion polymerization method), the grafting of aminoalkylcarboxylate and aminoalkylphosphonic ligands (*via* an intermediary amination step of PGMA) allows increasing sorption capacities due to the specific reactivity of carboxylate and phosphonate groups, giving iminodiacetate (IDA-PGMA) and iminodiphosphonate (IDP-PGMA), respectively. In the second step, functionalized-PGMA was ball-milled with pre-formed magnetic nanoparticles using high-energy planetary milling, resulting in a magnetic nanocomposite structure (M-IDA-PGMA and M-IDP-PGMA). These sorbents were characterized by elemental analysis, FTIR, XRD, pH_ZPC_, TEM, and VSM. The magnetic nanocomposite sizes were around 10.0 nm. The super paramagnetic properties of the hybrid materials make their solid/liquid separation quite easy using an external magnetic field. These materials were investigated for uranium sorption. Optimum pH was found to be close to 4.0; the maximum monolayer chemisorption capacities reach 122.9 and 147.0 mg g^−1^ for M-IDA- and M-IDP-PGMA, respectively. The adsorption activation energies were calculated from the Arrhenius equation. The sorption is spontaneous, endothermic and controlled by entropic change. Sorbents were tested for U(vi) removal from a real acidic leachate of ores collected in the El-Sella mining area. Finally, sodium bicarbonate revealed efficiency for uranium desorption and the re-use of sorbents was successfully tested for five cycles.

## Abstract 2

Designing and fabricating nanocomposite magnetic sorbents (with more accessible active sites for achieving high sorption capacities, selectivity and rapid kinetics) has become an impending challenge in the removal of radionuclides.

## Graphical abstract

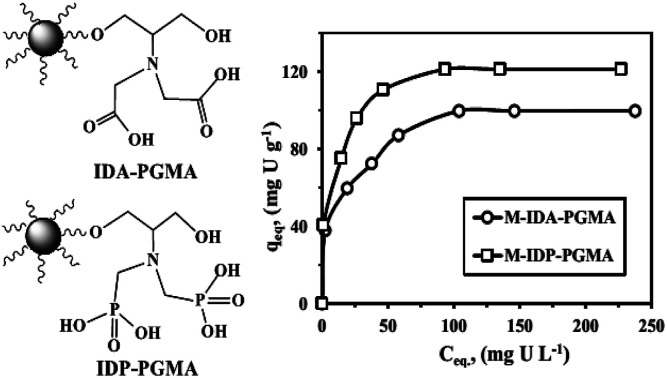

## 1. Introduction

Uranium is very important for nuclear energy production. However, its resources are rather limited and there is an expected shortage of uranium in the near future. Moreover, it is one of the most hazardous biotoxic radionuclides. Thus separation and recovery of uranium is a significant process from the viewpoints of both reasonable utilization of uranium resources and environmental protection.^1–4^ Sorption processes, including ion-exchange and chelating resins, represent an interesting method for the recovery of metal ions from dilute effluents due to their environmental friendliness and high efficiency.^1–4^ Various sorbents such as graphene oxide,^5^ organic-inorganic chelating sorbents,^4,7^ and activated carbon,^8^ have been used for uranium recovery from liquors. Chelating resins are polymers with reactive functional groups that chelate metal ions. The chelating properties roughly obey the Hard and Soft Acid Base theory (HSAB) developed by Pearson.^4–7^ Since uranium is a hard acid with a higher affinity towards hard bases, therefore, its chelating agents with O, N, and P groups are highly effective for the selective sorption of uranium ions (high capacity, selective separation).^4,10–12^

Grafting of reactive groups offers the possibility to improve sorption capacities, selectivity, and the pH range for efficient sorption.^4,6,7^ Reactivity may be changed with grafting new types of functional groups (such as carboxyl groups, alone or in combination with amine groups) through diethylenetriamine (DETA),^6^ or amino-acids.^7^ Moreover, organophosphorus compounds and their derivatives are known as good metal-complexing agents for industrial chemicals in water treatment, metal extraction, or pollution control especially uranium.^4,10–12^

Glycidyl methacrylate (GMA) is a reactive monomer, poses vinyl and epoxy functions, which meets the requirements for further post-chemically functionalization by a ring-opening reaction with one of several reactants, such as amines, carboxylic acids, *etc.*^10–13^ Attention has been focused on GMA, due to their abundance, renewability, and usually cost-effectively, so they can be used as product for post-chemically modification to enhance sorption efficiency.^9,13^

Nanocomposite is a multiphase material, for which one of them has nano-sized dimensions, especially magnetic-nanocomposites embedded with Fe_3_O_4_ nanoparticles, received a great attention since decreasing the particles sizes allows substantially reducing the limitations due to resistance to intraparticle diffusion and increasing specific surface area. Moreover, using an external magnetic field for recovering spent sorbents at the end of the sorption process.^14,15^ ESI† section reports the recent trends and developments of magnetic-nanocomposites in synthetic design and applications were explained in details, (Fig. SI(1)†). Sun *et al.*,^13^ prepared polymer core magnetic shell type structure by an *in situ* Fe_3_O_4_ coprecipitation into the pores of microspheres by an *in situ* Fe_3_O_4_ coprecipitation into the pores of polymer microspheres. On the other hand, magnetic-nanocomposite core–shell structures (*i.e.* magnetic core polymer shell type) were synthesized by encapsulation of homogeneously dispersed magnetic (Fe_3_O_4_) nanoparticles within the structure of polymer matrix (Fig. SI(1)†).^17,18^ For example, the one-pot hydrothermal precipitation of chitosan in the presence of Fe^2+^ and Fe^3+^ ions, *i.e.* Fe_3_O_4_ are synthesized inside polymer matrix.^7^ These techniques may have serious environmental drawbacks, complicated synthesis schemes and low yield that may reduce the materials efficiency. Thus, an alternative facile solid-state approach has been used to core–shell nanocomposite that has a lot of advantages such as simple to handle, reduce pollution, low cost, efficiency in mixing and dispersing of both inorganic and organic materials homogeneously and solvent free. In addition, it has the ability to produce large-scale quantities of powdery materials at room temperature.^16^ This technique consists of solid-state reaction between the reagents mixture or precursors using high-energy planetary ball milling. In this work, a new route for manufacturing of two new core–shell multi-functionalized polyglycidyl methacrylate (PGMA)-magnetic nanocomposites in two steps. First, PGMA immobilized with iminodiacetate, and iminodiphosphonate ligands obtained by a two-step grafting procedure: (a) first amination of the PGMA through treatment with ammonia solution, and (b) grafting of carboxylate and phosphonate groups on N-terminal functionality through reaction with monochloroacetic or phosphonic acid, giving iminodiacetate-PGMA (IDA-PGMA) and iminodiphosphonate-PGMA (IDP-PGMA). In the next step, magnetic nanocomposites were prepared using a facile solid-state method: by milling together pre-formed magnetic nanoparticles and functionalized PGMA. The prepared nanocomposites were characterized by CHNP analysis, FT-IR, pH_ZPC_, TEM, XRD, and VSM. Sorption properties for uranium are studied considering the effect of pH, uptake kinetics, sorption isotherms, and thermodynamic parameters. Regeneration and recycling of the sorbents were investigated for five cycles. Finally, sorbents testing the recovery of uranium from acidic mining effluent.

## 2. Material and methods

### 2.1. Materials

Glycidyl methacrylate (GMA), ammonia solution (33%), polyvinylpyrrolidone (PVP K-30), 2,2-azobisisobutyronitrile (AIBN), monochloroacetic acid, dimethyl-formamide (DMF), and FeSO_4_·7H_2_O and FeCl_3_ were purchased from Wako Chemical Co. Ltd. (Japan). Arsenazo III, and formaldehyde solution (HCHO, 37%) was obtained from Fluka (Buchs, Switzerland). Phosphorous acid was supplied by Sigma-Aldrich (Darmstadt, Germany). Uranium stock solution was prepared from uranyl acetate (UO_2_(OCOCH_3_)_2_·2H_2_O), supplied by Sigma-Aldrich. The salt was dissolved in concentrated H_2_SO_4_ under heating before being diluted in demineralized water (at final concentration: 1.0 g U L^−1^). Uranium concentration was determined using Arsenazo III colorimetric method,^17^ and spectrophotometer (Metertech Inc, model SP-8001).

### 2.2. Preparation of magnetite (Fe_3_O_4_) nanoparticles

Magnetic nanoparticles were prepared by a simple precipitation method of Fe^2+^ and Fe^3+^ ions by NaOH:^7^ by mixing of FeSO_4_ and FeCl_3_ with a molar ratio 1 : 2, respectively. The solution was chemically precipitated at 40 °C by addition of 2.0 M NaOH dropwise with constant stirring, under the protection of N_2_ gas at controlled pH (10.0–10.5), followed by hydrothermal treatment to obtain better magnetic properties, where the suspension was matured by heating at 80 °C for 1 h under continuous stirring. Finally, the precipitate was recovered by magnetic separation using super-magnets, and extensively washed with deoxygenated water (using N_2_ gas). Finally the prepared magnetite was dried at room temperature.

### 2.3. Synthesis polyglycidyl methacrylate (PGMA) derivatives

**Fig. 1:**
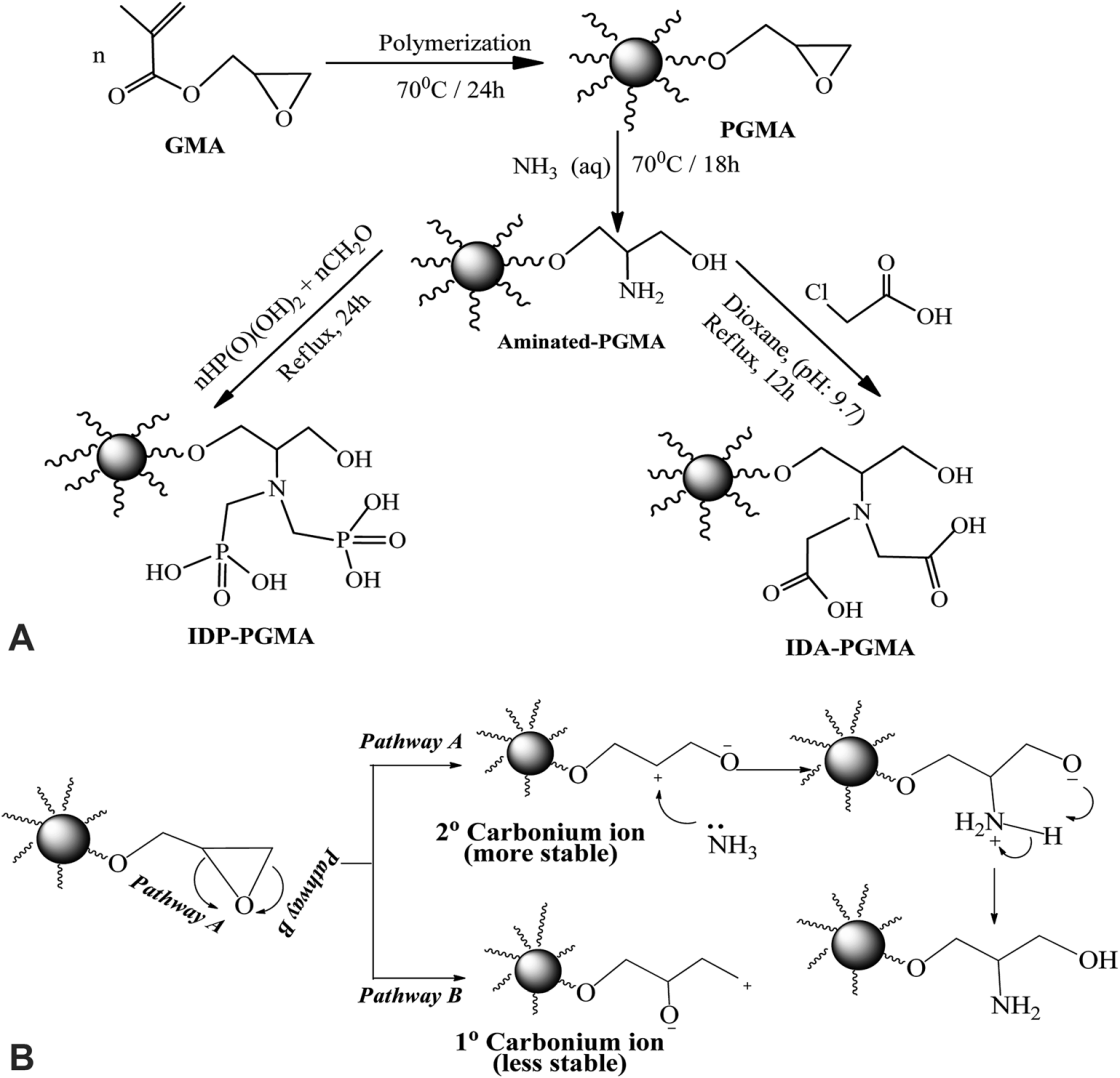
(A) Scheme for the synthesis of iminodiacetate and iminodiphosphonate-functionalized polyglycidyl methacrylate: (IDA-PGMA) and (IDP-PGMA), respectively. (B) Scheme for the synthesis of epoxy ring opening with ammonia.

Schematically illustration for the synthesis route and the chemical structure of IDA-PGMA and IDP-PGMA sorbents (Fig. 1A). The PGMA spheres were prepared using dispersion polymerization method as follows: dispersion medium (dissolving PVP K-30 (3.0 g)) into C_2_H_5_OH/water solution (90%, w/w) was prepared in a 250.0 mL four-necked flask. AIBN initiator (0.2 g) was added to the GMA monomer phase (10.0 g), then transferred into the dispersion medium, and the solution was then bubbled with N_2_ for 30 min. The mixture was refluxed under mechanical stirring (120 rpm) for 24 h at 70 °C.^9,13^ The product (i) PGMA was filtered off and repeatedly washed with ultrapure water and ethanol for several times, and dried under vacuum at room temperature. In the second step, PGMA (3.0 g) was dispersed in C_2_H_5_OH (20.0 mL), then ammonia (30.0 mL) was added. The suspension was stirred for 18 h under reflux at 80 °C:^6,7^ the product (ii) PGMA-NH_2_ was centrifuged and extensively washed. Finally, for grafting of phosphonic acid groups on the intermediary product (PGMA-NH_2_) through the reaction with phosphorous acid (3.0 g was dissolved in 50.0 mL of water and HCl (1 : 1, v/v)), then PGMA-NH_2_ (1.0 g) was added in three-necked flask (200.0 mL), the mixture was heated to reflux, and HCHO solution (7.5 mL) was added dropwise over 60 min, and the reaction was maintained under reflux for 24 hours.^9,18^ For grafting of carboxylic acid groups: the PGMA-NH_2_ (2.0 g) was suspended in DMF (25.0 mL), then monochloroacetic acid (10.0 g) was added. After suspension, the pH adjusting to 9.0–9.5, the mixture was stirred under reflux for 18 h.^6^ The final products were filtered, repeatedly washed with ultrapure water and ethanol for several times, and dried at 80 °C to give IDP-PGMA and IDA-PGMA sorbents.

### 2.4. Synthesis of core–shell nanocomposite

Magnetic-nanocomposite was prepared as core–shell structured material using high-energy ball-milling (planetary milling system, Fritsch Planetary Mills Pulverisette 7 classic line, Idar-Oberstein, Germany). Magnetite nanoparticles (0.5 g) were mixed with 1.0 g of functionalized PGMA (*i.e.*, 1 : 2 wt mass ratio). This amount of mixed solids (*i.e.*, 1.5 g) was equally distributed into YTZ working stations; the ball-to-powder weight ratio was set to 10 : 1 (YTZ balls, chemical composition (Y_2_O_3_–ZrO_2_): 95% ZrO_2_/5.6% Y_2_O_3_; diameter: 2.0 mm). Milling was operated at 700 rpm velocity for 60 min (including 1 min stop every 20 min). This treatment led to the manufacturing of core–shell magnetic nanocomposite.

### 2.5. Characterization of nanocomposites

Elemental analysis was investigated using an automatic analyzer Micro Corder JM10 (J-Science Lab Co., Japan). Phosphorus content was specifically analyzed after mineralization using sulfuric acid/nitric acid digestion.^19^ Fourier transform infrared (FTIR, JASCO-6600 spectrometer, Japan) was used in the wavenumber range 4000–400 cm^−1^ under normal conditions. The magnetic properties of nanocomposite samples were measured by using vibrating sample magnetometer at room temperature (VSM, 730T, Lakeshoper, USA). X-ray diffraction (XRD) were taken using a SmartLab X-ray Diffractometer (RIGAKU, Japan). The data were obtained in the range 2*θ* = 7–80° with Cu K_α_ radiation. The nanocomposite samples were performed by high resolution-transmission electron microscopy (HR-TEM JEOL-2100, Jeol, Japan). The point of zero charge (pH_PZC_) of the sorbent particles was determined by the pH-drift method: the sorbents were equilibrated under agitation for 24 h with a series of 0.1 M NaCl solutions with different initial pH values (pH_i_); the equilibrium pH (pH_eq_) values was recorded and the pH_PZC_ is the pH value corresponding to pH_i_ = pH_eq_.^4^ Moreover, the pH_ZPC_ analysis used to show the stability of theses nanocomposites under vigorous agitation and at different pHs (pH range from 1 to 9.6).

### 2.6. Metal sorption and desorption

Batch sorption experiments were carried out to investigate pH effect, uptake kinetics, sorption isotherms, metal desorption and sorbent recycling. To study the pH effect, the sorbent dose was 0.5 g L^−1^ at initial pH (pH_o_) from 1.0 to 6.0, the mixtures were agitated at 200 rpm for 12 h at room temperature; sorption isotherms were studied at initial metal concentration from 25.0 to 300.0 mg U L^−1^ with a pH at 4.0. Kinetic measurements were performed at different intervals and sorbent dosage was 0.5 g L^−1^. After equilibration and phase separation, the initial and equilibrium uranium concentrations in the supernatant were analyzed. The sorption capacity (*q*_eq_, mg g^−1^) at equilibrium was calculated according to equation: *q*_eq_ = (*C*_o_ − *C*_eq_)*V*/*m*, where, *C*_o_ and *C*_eq_, (mg L^−1^), are metal concentrations at initial and equilibrium, respectively; *m* (g) is the weight of sorbent and *V* (L) is volume of solution.

Uranium-loaded sorbents were mixed and stirred with NaHCO_3_ (0.25 M) for 1.5 h, at room temperature. The adsorption–desorption cycle experiments were continued for 5 times, after elution, the desorption efficiency (DE) (*i.e.*, DE = *C*_D_ × *V*_(L)_ × 100/*q*_d_ × *m*_d_) and regeneration rate (RE) (*i.e.*, RE = *q*_d_ × 100/*q*_e_); where *C*_D_ (mg U L^−1^) is the uranium concentration in the eluant, *V* (L) is the eluent volume, *q*_d_ (mg U g^−1^) is the sorption capacity each step before desorption experiment, *q*_e_ (mg U g^−1^) is the first cycle sorption capacity, and *m*_d_ (g) is the sorbent mass used in the desorption experiments.

Note: in order to verify the reproducibility in the synthesis of the sorbents several batches of the PGMA derivatives were prepared and independently characterized. Some sorption testes were duplicate. Average values are reported and the standard deviation was around 6%. Analyses were duplicated with standard deviation less than 4%. Some examples of reproducibility tests are presented at the end of the ESI† section.

## 3. Results and discussion

### 3.1. Synthesis and characterization of sorbents

Fig. 1A shows the schematic route for the sorbents synthesis that have been designed by functionalization with aminoalkylcarboxylate and aminoalkylphosphonic moieties. PGMA microparticles (<75 μm) were prepared by dispersion polymerization method. For epoxide ring opening by ammonia solution (NH_4_OH_(aq.)_ → NH_3_ + H_2_O at 60 °C), amino groups is grafted. The nucleophilic attack of nitrogen atom of NH_3_ to the three membered epoxy ring may involve one of the two pathways (A or B) depicted in Fig. 1B. Epoxy ring opening takes place at the internal carbon atom of the ring as shown for pathway A, for which the process gives more stable internal secondary carbonium ion. On the opposite hand, if the attack of the amine nitrogen takes place at the external carbon (less sterically hindered), a primary less stable carbonium ion is formed. Based on the aforementioned facts, pathway A is more favorable for primary aminated molecule (PGMA-NH_2_).

**Fig. 2:**
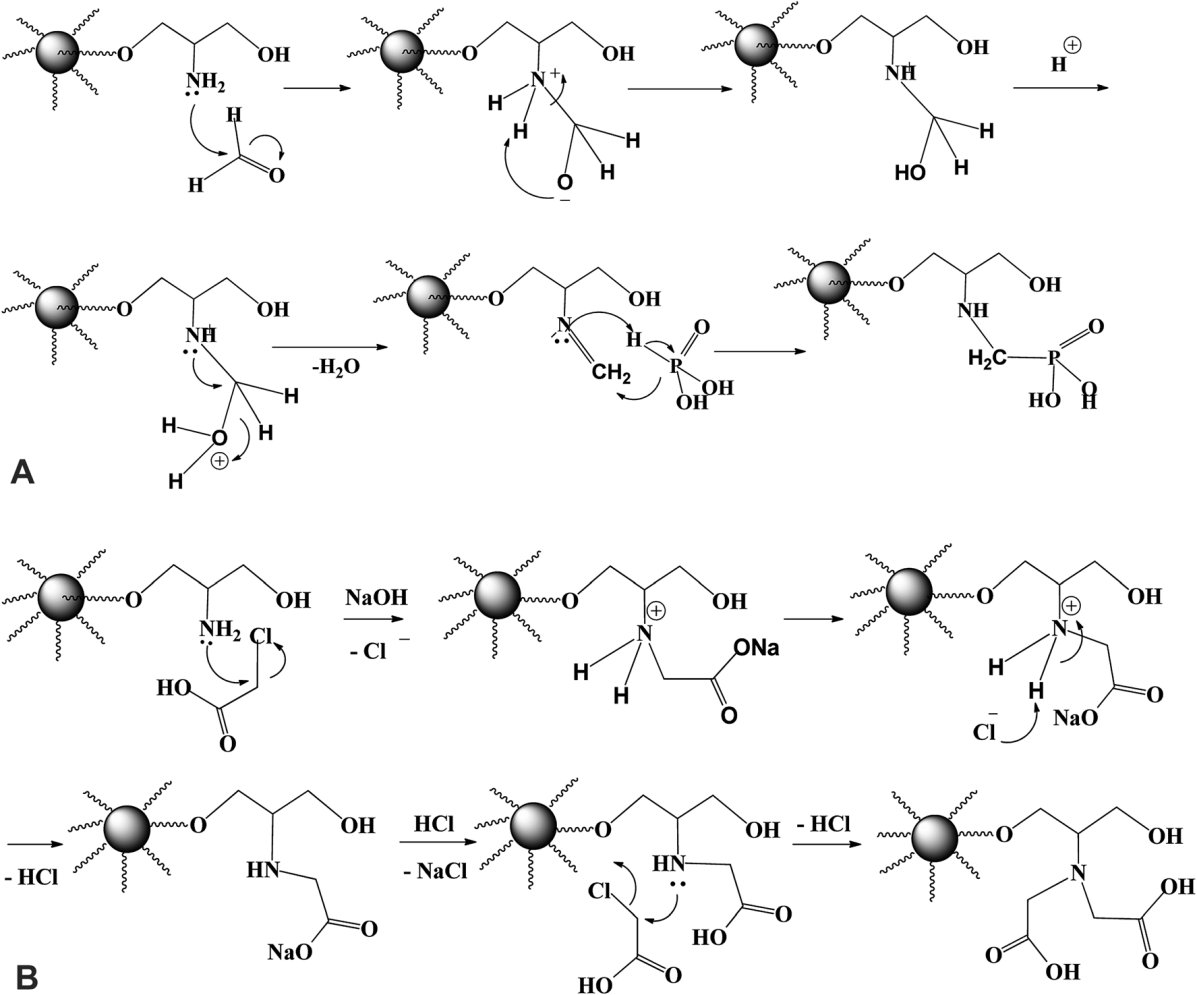
(A) Revised process for the *N*-phosphonomethylation of PGMA-NH_2_. (B) Revised process for the *N*-carboxymethylation of PGMA-NH_2_.

In the last step, methylene phosphonic groups are grafted on the intermediary product by reaction of phosphonic acid groups onto amine functions in the presence of formaldehyde, according to the general synthesis reaction (Fig. 2A, assuming the mechanism of *N*-phosphonomethylation). The primary amine group (–NH_2_) can be grafted by either one or two phosphonomethyl moiety for polysubstituted structure. In other words, the grafted polymer may have both mono-, and di-substituted amine (*e.g.* polymer –NH–CH_2_–PO_3_H_2_ and/or polymer –N(–CH_2_–PO_3_H_2_)_2_).^18,29^ In a similar manner, methylcarboxylation groups are grafted as condensation reaction of monochloroacetic acid with primary aminated molecule (PGMA-NH_2_) under basic conditions (Fig. 2B, assuming the mechanism of *N*-methylcarboxylation). HCl has released as a by-product resulting in decreasing the pH of the suspension from 9.7 to 4.9 at the end of the reaction.^6^ This was followed by grafting methylcarboxylate and methylphosphonate groups onto aminated molecule to produce to iminodiacetate- and iminodiphosphonate-functionalized PGMA, giving IDA-PGMA and IDP-PGMA respectively.

The magnetite fraction was determined by weight loss at 600 °C, to be about 33–34% of the total weight (*i.e.* one-third of the total weight of the final products is represented by the inorganic fraction).^20^ Several analytical techniques were used to investigate and describe the reactive groups.

#### 3.1.1. Elemental analysis

Materials elemental analysis in mass percentage at the different synthesis stages are presented in Table 1. Based on the theoretical structure of PGMA, the conversion of weight percentages for C, H and N elements in molar units means that the polymer can be approached by the heptameric formula: (C_7_H_10_O_3_)_7_.^9^ After amination and grafting of amine group (–NH_2_), the nitrogen content efficiently increased and reached 2.56 mmol N g^−1^ (mass percentage: 3.59%). After phosphonomethylation and carboxymethylation reaction of the aminated intermediary product (PGMA-NH_2_); demonstrated by the decrease of nitrogen content from 2.56 to 1.41 and 1.63 mmol N g^−1^ molar concentrations in IDP- and IDA-PGMA, respectively (mass percentages: from 3.59 to 1.97 and 2.28%, respectively): the molecular weight of the final derivatives strongly increase and then “dilute” N element in the final products. The simultaneous decrease of C and H mass fraction: C content drastically decreases before and after phosphonomethylation and carboxymethylation grafting, to a level comparable to C content in PGMA-NH_2_.^6^ This shows the efficient grafting of the phosphonic and carboxylic acid groups and demonstrates that reactions are almost quantitative. After incorporation of magnetite, phosphorus content changes from 1.75% (w/w) (*i.e.* 0.57 mmol P g^−1^) to 1.15% (*i.e.*, 0.371 mmol P g^−1^), *i.e.* P weight ratio in magnetic and non-magnetic product is about 66.0% (P content was reduced by 34.0% in magnetic nanocomposite). The same results were observed with decrease of C and H mass fraction which were about 66.0% in polymeric nanocomposite, this means that the organic polymer percent is about two-thirds rather than one-third for magnetic weight percent. This is in an agreement with the results obtained from thermal analysis for which the remaining is about 33.4%.

**Table:** Elemental analysis of the products at different stages of the process

Material	C (%)	H (%)	N (%)	P (%)	O^a^ (%)	pH_ZPC_
PGMA	57.80 (±0.02)	7.26 (±0.01)	0.31 (±0.03)	—	34.63	5.90
PGMA-NH_2_	49.68 (±0.04)	7.74 (±0.07)	3.59 (±0.02)	—	38.99	7.47
IDA-PGMA	42.97 (±0.05)	7.51 (±0.06)	2.28 (±0.01)	—	47.24	—
IDP-PGMA	40.86 (±0.08)	6.93 (±0.03)	1.97 (±0.04)	1.75 (±0.03)	48.49	—
M-IDA-PGMA	28.51 (±0.02)	4.73 (±0.04)	1.54 (±0.04)	—	—	3.45
M-IDP-PGMA	26.94 (±0.04)	4.44 (±0.02)	1.23 (±0.04)	1.15 (±0.04)	—	4.52

a Obtained by difference to 100% (w/w fraction). —: not detected.

#### 3.1.2. FTIR analysis

**Fig. 3:**
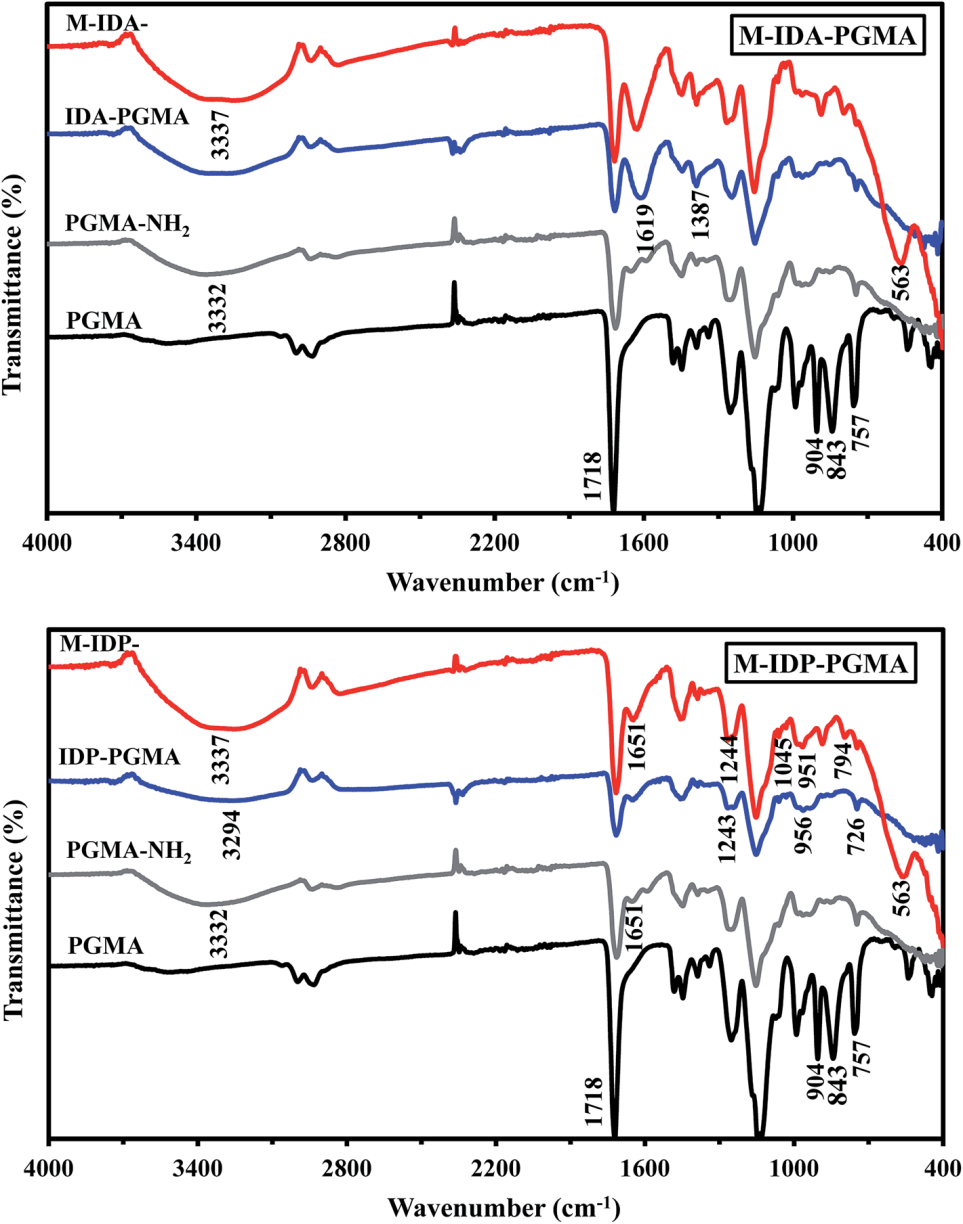
FTIR spectra of the products at different stages of the synthesis process.

FTIR spectrometry used to confirm the presence of the functional groups (Fig. 3). The peak at and around 563 cm^−1^ is assigned to stretching (Fe–O) vibration of Fe_3_O_4_ in both magnetic nanocomposites spectra.^2,20^ The FTIR spectrum of PGMA show strong peaks used for identifying the fingerprint of the polymer like: (a) at 1718 cm^−1^ ascribed to the carbonyl groups (*ν*(–COO–)) vibrations,^7,20,24^ and (b) at 843 and 904 cm^−1^ are the characteristic peaks assigned to asymmetrical expansion and stretching vibrations of the oxirane ring, respectively,^14^ and a peak close to 757 cm^−1^ assigned to epoxy ring.^9,21^ The peaks at the range 1300–1100 cm^−1^ are the stretching peaks of C–O vibrations.^7,20,22^ The PGMA-NH_2_ spectrum shows a large broad band appears around 3360 cm^−1^: associated to the combination of stretching of –OH, N–H groups, and inter H-bonds vibrations.^5–8^ The disappearance of the peaks associated to epoxy ring vibrations,^9,13,21^ and new bands at 1644 cm^−1^ and 1585 cm^−1^, attributable to N–H vibrations in 1° and 2° amines, respectively.^6–8^

IDP-PGMA spectrum shows the appearance of typical bands at 746, and 956 cm^−1^ assigned to *ν*(–P=O), and P–O–C stretching or to P–OH stretching,^4,10,18^ and at 1045 and 1244 cm^−1^, associated to P–O–R bond and P=O bond.^9,18,23^ IDA-PGMA spectrum shows the enlargement at 1639 cm^−1^, and the increase in the intensity of the peak at 1387 cm^−1^, probably be assigned to carboxylate groups.^6,7^ Lactone sixth membered ring (C=O) shows strong peaks at 1710–1740 cm^−1^. Moreover, the peak appearing at 1320 and 1065 cm^−1^, belongs to stretching of primary and secondary –OH groups vibrations, respectively.^7,20^

#### 3.1.3. XRD analysis

The XRD patterns was used to investigate the functionalized polymer-Fe_3_O_4_ nanocomposites (Fig. SI(2)†). The pattern change of the PGMA (poorly crystalline structure) which is characterized by two shoulders at around 2*θ* angles: 18° and two other weak shoulders at around 2*θ*: 30° and 38°.^9^ This XRD fingerprint, typical from PGMA materials is strongly affected after physical modification of the final materials with magnetite (Fe_3_O_4_). The incorporation of a magnetic core in the core–shell nanoparticles is confirmed by the appearance of eight characteristic peaks at 2*θ* = 18.33, 30.15, 35.52, 43.16, 53.55, 57.09, 62.69, 74.17. These peaks are associated with the following indices: (111), (220), (311), (400), (422), (511), (440), and (533), which are clearly representative of the spinel structure of magnetite (*i.e.*, Fe_3_O_4_).^14,20^ The size of the Fe_3_O_4_ crystallites was estimated from the Debye–Scherrer equation (*D* = *kλ*/*β*_1/2_ cos *θ*),^27^ where *D* is the average diameter of nanoparticles (Å), *k* is the Scherrer constant (*k* = 0.9), *λ* is the wavelength of X-ray radiation (1.5418 Å), *θ* is the diffraction angle, *h* is the angle of diffraction, and *β*_1/2_ is the full width at half maximum of X-ray diffraction peaks. The crystallite sizes were about 10.0 nm (using the (311) index at 2*θ* = 35.4°).

#### 3.1.4. Magnetic properties

Typical magnetization loops (hysteresis loop) (Fig. SI(3)†), the absence of remanence and coercivity, prove that these nanocomposites are superparamagnetic materials.^20,25^ The saturation magnetization of M-IDA- and M-IDP-PGMA nanocomposites were about 22.56 and 21.14 emu g^−1^, respectively; that easily recovered with the help of an external magnetic field. These values are much lower than the values obtained with pure (*i.e.*, 50–70 emu g^−1^) and significantly lower than the bulk phase Fe_3_O_4_ magnetic nanoparticles (*i.e.*, 92 emu g^−1^).^25,26^ This decrease can be explained by several factors including experimental conditions used for the synthesis of magnetic particles, nanometric size effect, particle crystallization and the amount of magnetite embedded in diamagnetic polymer layer *etc.*^7,25,26^

#### 3.1.5. TEM analysis

**Fig. 4:**
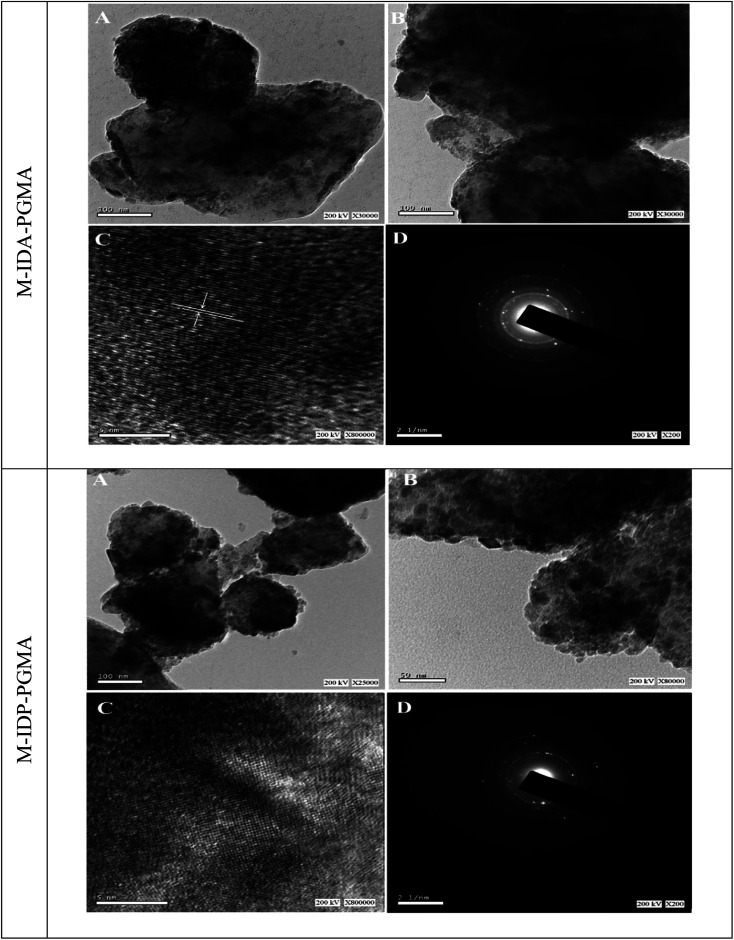
(A and B) are the TEM captures of the sorbent nanocomposite; while (C and D) are crystal lattice and SAED of the nanocomposite, respectively.

Nanocomposites morphological features were characterized by TEM. Fig. 4 shows that the nanocomposites have a spherical and homogeneously distribution and they can be described as monodisperse; however, the iron oxide Fe_3_O_4_ nanoparticles tended to aggregate, probably due to dipole/dipole magnetic attraction.^7,20^ The Fe_3_O_4_ nanoparticles are still in the nano-size level (<20 nm) confirming that; these materials can be considered as a nanocomposite.^14,15^ Moreover, the overall structure (average diameter 40–50 nm) shows different areas: the dark areas represent crystalline Fe_3_O_4_ with an average diameter of 10 nm, embedded into the bright ones are associated with organic framework polymer due to the different electron-absorbing abilities (*i.e.* the electron binding ability of Fe_3_O_4_ is higher than that of the polymer shell).^28^ Therefore, the cores can be identified as the darker region compared to the shell area. The crystallinity of nanomaterials was studied through the analysis of selected area electron diffraction (SAED). The SAED illustrated the polycrystalline nature and the rings are sharp and continuous, with high purity of the nanocomposites.

#### 3.1.6. pH_ZPC_

The pH_ZPC_ data were used to confirm the successful grafting of different functionalities such as amines, carboxylic, and phosphonic acids groups. As well as the effective charge surface of sorbents *via* measuring pH_ZPC_ values at each stage during the course of the reaction steps using the pH-drift method (Fig. SI(4)†). It is worth to note that incorporation of basic amino group into the polymer skeleton increases the pH_ZPC_ from 5.9 to 7.5 which is attributed to the p*K*_a_ value: 9.21 of the amino groups in free ammonia, and primary amines.^31^ While, further derivatization of amino group by carboxylic and phophonic acid groups through methylcarboxylation and methylphosphonation reactions, strongly influence the pH_ZPC_ values to be: 3.45 and 4.52 for M-IDA- and M-IDP-PGMA, respectively. The significant decrease in the pH_ZPC_ confirms that high degree of the amine substitution which is attributed to the decrease of the electron density around nitrogen atom of amino groups due to withdrawing power of <svg xmlns="http://www.w3.org/2000/svg" version="1.0" width="10.400000pt" height="16.000000pt" viewBox="0 0 10.400000 16.000000" preserveAspectRatio="xMidYMid meet"><metadata> Created by potrace 1.16, written by Peter Selinger 2001-2019 </metadata><g transform="translate(1.000000,15.000000) scale(0.011667,-0.011667)" fill="currentColor" stroke="none"><path d="M80 1160 l0 -40 40 0 40 0 0 -40 0 -40 40 0 40 0 0 -40 0 -40 40 0 40 0 0 -40 0 -40 40 0 40 0 0 -40 0 -40 40 0 40 0 0 -40 0 -40 40 0 40 0 0 -40 0 -40 40 0 40 0 0 80 0 80 -40 0 -40 0 0 40 0 40 -40 0 -40 0 0 40 0 40 -40 0 -40 0 0 40 0 40 -40 0 -40 0 0 40 0 40 -40 0 -40 0 0 40 0 40 -80 0 -80 0 0 -40z M560 520 l0 -40 -40 0 -40 0 0 -40 0 -40 -40 0 -40 0 0 -40 0 -40 -40 0 -40 0 0 -40 0 -40 -40 0 -40 0 0 -40 0 -40 -40 0 -40 0 0 -40 0 -40 -40 0 -40 0 0 -40 0 -40 80 0 80 0 0 40 0 40 40 0 40 0 0 40 0 40 40 0 40 0 0 40 0 40 40 0 40 0 0 40 0 40 40 0 40 0 0 40 0 40 40 0 40 0 0 80 0 80 -40 0 -40 0 0 -40z"/></g></svg> C=O and P=O functionality.^30,31^ The difference in the value of the pH_ZPC_ for the two sorbents can be directly associated to the strength of acid–base properties of the carboxylic and phosphonic acid groups.

Analysis of acid–base properties of α-amino acids (M-IDA-) compared with α-aminophosphonic acids (M-IDP-) showed that the stronger acid group of M-IDA with a pH_ZPC_ value lower than that of acid group in M-IDP-. This result is inconsistence with previously reported data which states that: the α-aminophosphonic considered as a stronger diacid with p*K*_a1_ 0.5–1.5 and p*K*_a2_ 5–6 compared to the acidity of mono-carboxylic acid close to 2.2–3.^30,31^ This difference is due to the degree of substitution of the phosphonic acid groups compared to the carboxylic acids ones. Ongoing to steric hindrance effect, as the phosphorous atom has much larger atomic radius than carbon,^32,33^ the steric effect of phosphorus hinder the formation of N-bearing disubstituted phosphorylated material, namely phosphomethylation reaction is a mono-substituted while in carboxymethylation reaction is di-substation reaction.

### 3.2. Uranium sorption properties

#### 3.2.1. Effect of pH

**Fig. 5:**
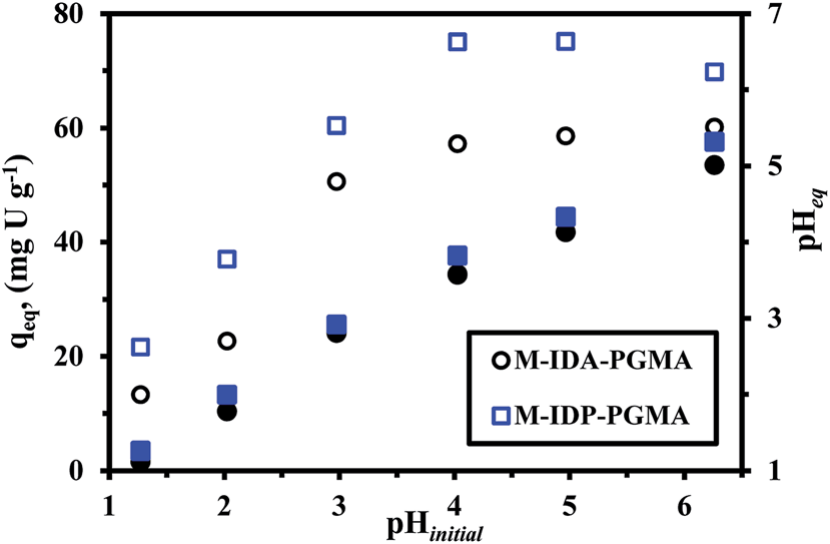
Effect of pH on U(vi) sorption: sorption capacity *versus* initial pH (open symbols) and equilibrium pH *versus* initial pH (closed symbols) (*C*_o_: 50 mg U L^−1^; *T*: 26 °C; SD: 0.5 g L^−1^; time: 12 h).

The solution pH plays a key role to the affinity of sorbents for target metals (herein U(vi) ions). Below pH 1.25, the sorbent magnetic core (Fe_3_O_4_) may partially dissolve, and lose stability, while at and above pH 6; the precipitation of uranyl colloidal ions UO_2_(OH)_2_·H_2_O species may begin and be formed.^20,33^ Effect of pH on the sorption capacity of U(vi) was investigated in pH range of 1.25 to 6.25. Fig. 5 shows the effect of initial pH value on the U(vi) sorption. Sorption capacity increases for both sorbents from pH 1.27 to pH 4.03; this was followed by, a very little increase or nearly a plateau for M-IDA-PGMA, meanwhile, for M-IDP-PGMA, a slightly decreased after reaching the maximum.

The sorption capacity progressively increases for M-IDA- (57.3 mg U g^−1^), on the other hand, a sharper increase in sorption capacity is observed at pH 4.0–5.0 for M-IDP-PGMA (75.1 mg U g^−1^). The main differences between the both nanocomposites, in the effective sorption sites *e.g.* carboxylate and phosphonate groups, and also, may be due to the lactone sixth membered ring of M-IDA-PGMA formation (Fig. SI(5)†). These results are consistent with previous comments on the chemistry of reactive groups and metal species. These sorption behaviors are probably due to the effective deprotonation of reactive groups: carboxylate, phosphonate, and amine sites; that will be able to bind metal cations: this can be correlated to the significant increase in sorption capacity.^9,10,14^ Results could be correlated to the pH_ZPC_ values: 3.45 for M-IDA-against 4.52 for M-IDP-PGMA; as a consequence, the surface of the sorbents is negatively charged, is expected to be more favorable to uranium sorption.

Moreover, as the affinity of uranyl species for sorbent may also change as a function of the pH value due to the change in metal speciation. In acidic solutions (below pH 4.0), divalent free UO_2_^2+^ ions predominate in the solution. When the pH increases (range 4.0–5.5), several cationic mononuclear or polynuclear species appeared such as: UO_2_(OH)^+^, (UO_2_)OH^+^, and (UO_2_)_2_(OH)_2_^2+^,^7,33^ while in the presence of an excess of sulfate (SO_4_^2−^) anions, UO_2_(SO_4_)_2_^2−^, anionic specie appeared and the fractions of free uranyl and neutral uranyl sulfate progressively decreased.^4^ For these reasons, taking into account optimum efficiency, pH variation and both the stability of the sorbent and the speciation of metal ions, further experiments were performed at initial pH 4, after sorption, the pH stabilizes (pH_eq_) in the range 3.6. It is noteworthy that the equilibrium pH (pH_eq_) of uranium solutions after sorption were reported since the initial pH may change during sorption process for both sorbents which may be explained due to the acid–base properties of each sorbent. The pH change (ΔpH) is more marked in the case of M-IDA-PGMA, the pH variation is negligible (<0.3 pH unit), while for the pH range 1–3 and above the pH increases by approximately 0.5–0.9 unit in the range 4–5 and strongly affected by about 1.3 unit at pH 6 (this is probably associated to proton exchange and uranium ions binding). The pH change is less marked in the case of M-IDP-PGMA, for pH < 3 (about <0.1 unit). An increase by 0.6 unit in the range 4–5, and about 1 pH unit at pH 6. This is probably associated to the degree of grafting of amino-methylcarboxylate and amino-methylphosphonic functional groups. This explanation reported in details above in pH_ZPC_.

#### 3.2.2. Uptake kinetics

**Fig. 6:**
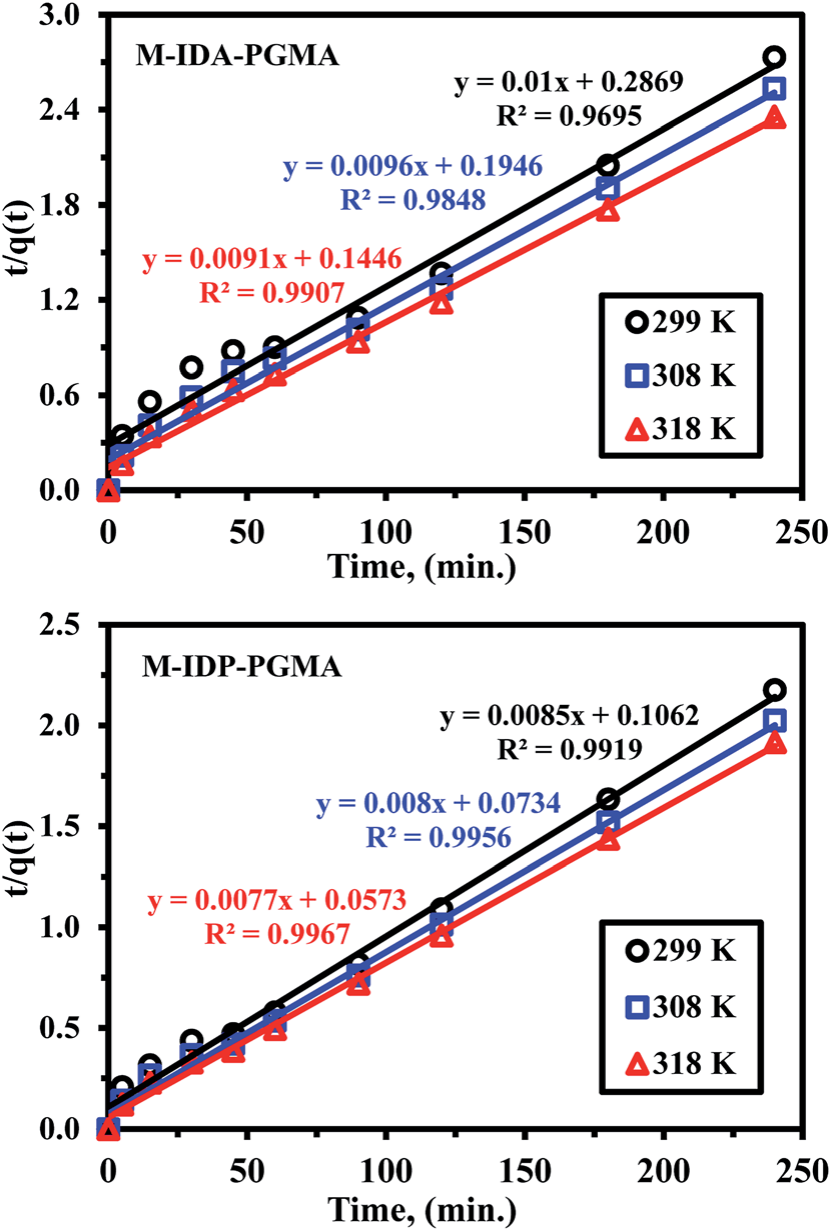
Uptake kinetics plots for U(vi) sorption using M-IDA-PGMA and M-IDP-PGMA sorbents at different temperature (pH: 4.02; *C*_o_: 100 mg L^−1^; SD: 0.5 g L^−1^).

Uptake kinetics for uranyl ions were compared for the two sorbents at different temperatures (Fig. 6). All the curves, regardless sorbent type and temperature, show similar trend. Initially, a very fast initial sorption (for the first 30 min) represents 54–69% of total sorption with an almost linear increase in sorption capacity (*vs.* time), (corresponding to a physisorption mechanism), and decreased slowly with increasing reaction time (*i.e.* followed by a chemical sorption involving charge neutralization, coordination, and chelation till equilibrium). After that, sorption rate was relatively nearly zero due to the attainment of the equilibrium.^4,34,35^ The change in the rate of uranyl ions sorption depends mainly on the accessible sorption sites located at the surface of sorbent particles, the saturation degree of these active sites (*e.g.* amine, carboxylic and phosphonic acid groups) and the sorption velocity is enhanced by the high concentration gradient between the solution, surface and the internal reactive sites.^7,9,34^ By increasing sorption time, sorption velocity decreases due to a decrease in the concentration gradient and to the contribution of the mechanism of resistance to intraparticle diffusion.^35–37^ At the end, the equilibrium plateau is systematically due to the availability of free active sites strongly decreased; where most of them being occupied, approached saturation and the rate of uranyl ions sorption became zero value due to adsorption–desorption equilibrium^34–36^ reached within 120 and 90 min of contact for M-IDA- and M-IDP-based sorbents, respectively. Preliminary studies have shown that an extended contact time (extended up to 12, and 24 h) does not significantly change sorption performance. Thus, 120 min of a contact time is sufficient to almost achieve the equilibrium. These trends are confirmed by the Weber and Morris plots (Table SI(1) & Fig. SI(6)†) for which different diffusion behaviors. The first portion with steep slope represented external surface adsorption or instantaneous adsorption stage, corresponds to the binding of uranyl ions on the sorption sites that are free and available at the surface (or within the first external layers) of sorbent particles. The second portion was the gradual adsorption stage (diffusion in micropores, mesopores and micropores), where the intraparticle diffusion was rate-controlled and started to slow down due to the low metal concentration in solution.^39,40^ Also, suggesting that the progressive saturation of external layers of the sorbent with uranyl species or hydrolyzed uranyl species with the largest ionic size (see above) may contributes to both hindered diffusion and slow kinetics, and to changing the diffusion performance in the polymer leading to these multi-linear plots.^34^ It is noteworthy that the values of intraparticle diffusion rate constants: both first and second rate constants (*K*_id,1_ & *K*_id,2_) were larger values, for M-IDP- and M-IDA-sorbent, respectively, and especially, having the following sequence of M-IDP- > M-IDA-sorbent, implying faster sorption processes for M-IDP-sorbent. The third rate constants (*K*_id,3_) values were nearly relatively close to zero for different sorbents supposing the attained equilibrium state.^36,37^

Furthermore, uptake kinetic has been modeled using the pseudo-second order rate equation (PSORE) and different temperatures (Table SI(1)†).^38^ Plotting *t*/*q*(*t*) *versus* time describes the kinetic profiles for PSORE (Fig. SI(7)†) and the parameters are summarized in Table 2. The observed higher values of both experimental values of *q*_eq,(exp.)_ (mg U g^−1^) and the overall rate constant (*K*_2_; mg g^−1^ min^−1^) with increasing temperature may be related to the dehydration effect of both active sites and metal ion facilitating the adsorbent/metal ion interaction, regardless of the sorbent type. The value of the calculated equilibrium sorption capacities is consistent with the experimental observation (*i.e.* a satisfactory agreement was obtained between calculated *q*_eq,(cal.)_ and experimental values of *q*_eq,(exp.)_) (Table 2), and were systematically overestimates the maximum *q*_eq,(exp.)_. According to pseudo-second order kinetics, U(vi) sorption was chemisorption reaction involving valence forces for sharing or exchanging electrons through complexation, coordination and chelation between sorbent surface and metal.^7,33,35^

Kinetics parameters for U(vi) sorption

**Table:** 

Sorbent	Temp. (K)	PSORE		
		*K*_2_ × 10^−5^ (mg g^−1^ min^−1^)	*q*_eq_ (mg U g^−1^)	*R* ^2^
M-IDA-PGMA	299	34.86	100.00	0.970
	308	47.36	104.17	0.981
	318	57.27	109.89	0.987
M-IDP-PGMA	299	68.03	117.65	0.992
	308	87.19	125.00	0.996
	318	103.47	129.87	0.997

**Table:** 

Sorbent	Temp. (K)	sRIDE		
		*K*_id,1_ (mg g^−1^ min^−0.5^)	*K*_id,2_ (mg g^−1^ min^−0.5^)	*K*_id,3_ ( mg g^−1^ min^−0.5^)
M-IDA-PGMA	299	0.0544	0.0463	0.00005
M-IDP-PGMA	299	0.1422	0.0561	0.00020

#### 3.2.3. Sorption isotherms and thermodynamic parameters

**Fig. 7:**
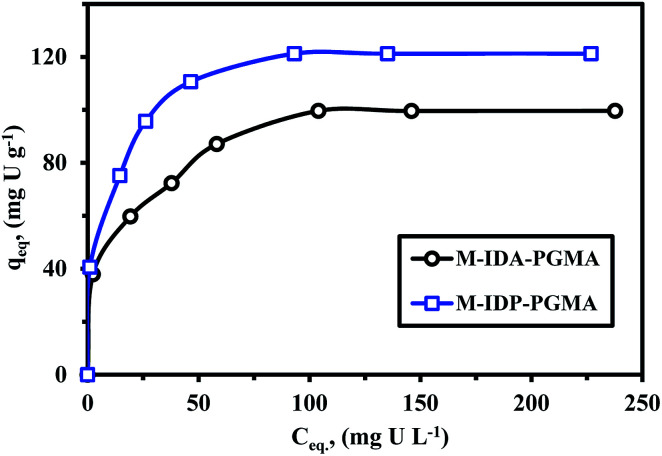
U(vi) sorption isotherms using M-IDA-PGMA and M-IDP-PGMA sorbents (pH: 4.06; *T*: 26 °C; SD: 0.5 g L^−1^; time: 12 h).

To evaluate sorbents capacities, uranium sorption isotherms were determined at pH 4.0 and 299 K (Fig. 7). Sorption capacity was increased with increasing the initial and equilibrium uranium concentrations. Sorption curves are generally following the same trend by a number of successive steps including: (a) a sharp initial section with strongly increasing sorption capacity followed by (b) a progressive increase in the sorption capacity, and (c) terminated by a saturation plateau. As the initial uranium concentration gradually increases, and the driving force become more strongly, providing enough more energy between the solution and the active sorption sites.^34–37^ The sorption progressively increases to reach a plateau close to maximum sorption capacities (*q*_max_) reach 99.6 and 121.2 mg U g^−1^ for M-IDA- and M-IDP-PGMA, respectively.

ESI† section reports the main equations used for modeling the isotherm profiles, including the Langmuir, Freundlich, and the Dubinin–Radushkevich (D–R) isotherm model (Table SI(1)†).^9,35^ Different models parameters (with their determination coefficients, *R*^2^) are summarized in Table 3. Adjustment quality can be evaluated by the value of *R*^2^ and by comparison of the calculated and experimental values of equilibrium sorption capacities (Table 3). Fig. SI(8)† shows the comparison of simulated curves for the Langmuir and Freundlich models. Obviously the Langmuir equation fits the experimental data much better than the Freundlich equation for which *R*^2^ was systematically higher than 0.99 (compared to less than 0.98 for Freundlich), this was expectable based on the shape of sorption isotherms: the saturation plateau is consistent with the asymptotic trend associated to the Langmuir equation, while the Freundlich equation which is a power-like function.^4,35^

Isotherm constants for U(vi) sorption

**Table:** 

Sorbent	Langmuir isotherm constants		
	*q*_max_ (mg U g^−1^)	*K*_L_ (L mg^−1^)	*R* ^2^
M-IDA-PGMA	103.09	0.1273	0.996
M-IDP-PGMA	123.46	0.2275	0.998

**Table:** 

Sorbent	Freundlich isotherm constants		
	*n*	*K*_F_ (mg g^−1^)	*R* ^2^
M-IDA-PGMA	4.03	30.13	0.986
M-IDP-PGMA	4.22	41.33	0.978

**Table:** 

Sorbent	D–R isotherm constants		
	*q*_max_ (mg U g^−1^)	*E*_DR_ (kJ mol^−1^)	*R* ^2^
M-IDA-PGMA	95.98	8.17	0.9164
M-IDP-PGMA	120.52	9.05	0.9584

It is noteworthy that the modeled values for equilibrium sorption capacities (*q*_eq_) were consistent with experimental observations and were systematically underestimated using the Freundlich (the differences about 66.2–69.8%) and overestimated by the Langmuir model (the differences do not exceed 1.2–3.5%). The simulated curve is close to experimental points as a confirmation of the suitability of the Langmuir equation to fit sorption isotherm. This suggests (to be verified by appropriate analytical procedures) that metal sorption occurs through monolayer uniform sorption, with a finite number of identical sites distributed over the sorbent surface.^7,33,35^

For favorable analysis sorption properties, the value of a dimensionless constant (*R*_L_ = (1 + *K*_L_*C*_o_)^−1^): where *K*_L_ is the Langmuir constant and *C*_o_ is the initial metal concentration; must be lower than one.^35,36^ All *R*_L_ values for the sorbent were lied between 0.03 and 0.27 for M-IDP- and between 0.06 and 0.46 for M-IDA-sorbent; all of them being smaller than 1, this means that uranyl sorption on both sorbents is highly favorable, regardless of metal concentration. The Dubinin–Radushkevich gives information on the mean sorption energy (*E*_DR_) that is usually employed for discriminating systems driven by physical or chemical sorption.^38,40^ Fig. SI(8)† shows the D–R mathematical fit of experimental profile, where constants (such as *q*_max_, and *E*_DR_) are reported in Table 3. The mean sorption energy (*E*_DR_, kJ mol^−1^) corresponds to the free energy exchanged for the transfer of one mole of solute from infinity (in solution) to the surface of the sorbent. *E*_DR_ values were systematically above 8.0 kJ mol^−1^, this is usually associated to chemisorption mechanism. This is also consistent with the better fit of kinetic profiles by the pseudo-second order rate equation (which is usually associated with chemical sorption).

#### 3.2.4. Sorption activation energy

Sorption activation energy of U(vi) was calculated using Arrhenius equation aiming at assessing the sorption nature (physical or chemical): (ln *k*_2_ = ln *k*_o_ − (*E*_a_/*RT*)),^38,39^ where *k*_2_ is the overall rate constant of the PSORE (g mg^−1^ min^−1^), *R* is the gas constant (8.314 J mol^−1^ K), *k*_o_ is the Arrhenius constant which is a temperature independent factor (g mg^−1^ h^−1^), *E*_a_ is the activation energy of sorption (kJ mol^−1^).

**Fig. 8:**
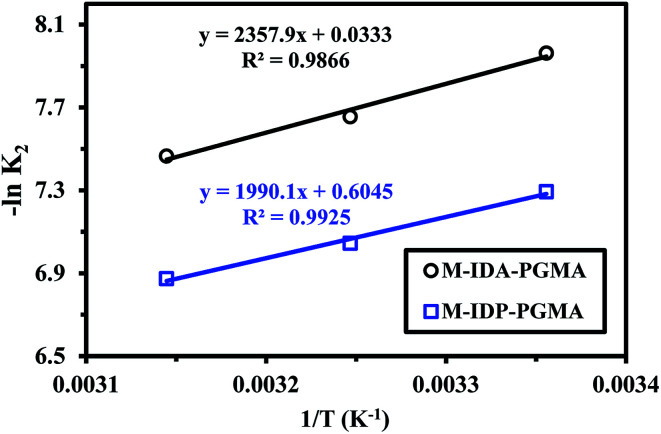
Arrhenius plots of ln *K*_2_*vs.* 1/*T* of U(vi) sorption using M-IDA-PGMA and M-IDP-PGMA at different temperature (pH: 4.02; *C*_o_: 100 mg L^−1^; SD: 0.5 g L^−1^).

Plotting −ln *k*_2_*versus* 1/*T* gave a straight line with a slope of (−*E*_a_/*R*) (Fig. 8). The apparent activation energies for U(vi) sorption were found to be 19.59 and 16.55 kJ mol^−1^ for M-IDA- and M-IDA-PGMA, respectively. The lower activation energy of M-IDP-PGMA than of M-IDA-PGMA indicates faster sorption kinetics of M-IDP- as reported above. Again, the lower values of activation energy confirm that the sorption process is controlled by interaparticle diffusion.^38,39^ Calculation of activation energy (*E*_a_) was used to determine the type of adsorption. It is known that activation energy ranging from 5 to 40 kJ mol^−1^ characterizes physisorption process, while chemisorption has higher activation energy (40–80 kJ mol^−1^). Generally, physical sorption process was a multilayered, quick and reversible process controlled by the van der Waals force, therefore, little energy was required. The chemical sorption was monolayered, slow and process controlled by chemical bonds, therefore, larger activation energies are required. In addition, both processes may exist together.^38,39^ The mean activation energy (*E*_a_) of the U(vi) ions for both sorbents are systematically below 40 kJ mol^−1^, showing that sorption proceeds through a physisorption mechanism.

Conclusions coming from uptake kinetics, sorption isotherms and adsorption activation energy sound to be contradictory. The PSORE model is generally associated to a chemisorption process (controlled by the mechanism of electron sharing, or exchange between sorbent surface and sorbate), and the mean sorption energies (*E*_DR_) (being more than 8 kJ mol^−1^) come along with chemisorption process, while a typical of physisorption, which matches with low sorption activation energy (5 to 40 kJ mol^−1^) determined from Arrhenius equation. This suggests a dual mechanism of physisorption (electrostatic forces) and chemisorption (ionic forces: coordination) owing to heterogenous binding sites in M-IDA-PGMA and M-IDP-PGMA.^40^

Table 4 reports uranium sorption capacities of a series of different sorbents. Since the experimental conditions are not identical (*e.g.* sorbent dosage, time, pH, and solution composition), a direct comparison is not easy. These nanocomposites (M-IDA- and M-IDP-PGMA) have comparable in terms of two parameters (sorption capacities and uptake kinetics) to other sorbents (though some materials such as magnetite nanoparticles,^41^ modified graphene oxide,^40^ amino acid derivatives of magnetic chitosan nanoparticles^7^ and synthetic resins *e.g.* Amberltie IRA-910 resin^34^ show greater sorption performance). Remarkable sorption levels were reported with polyaminated sorbents *e.g.* tetraethylenepentamine (TEPA) modified glycidyl methacrylate magnetic,^42^ TEPA-magnetic chitosan,^43^ organo-phosphorus sorbents, *e.g.* aminophosphonate functionalized chitosan sorbents,^4^ phosphonate-,^44^ and phosphonate and amino-groups,^45^ functionalized mesoporous silica, however, their pH range of application is generally larger (lower decrease in sorption performance at low pH).

**Table:** Comparison of sorption capacity for U(vi) with various sorbents

Sorbents	Equilibration time (min)	*q*_max_ (mg U g^−1^)	pH	Ref.
Magnetite nanoparticles	360	5.5	7	41
CS-Me and CS-Ph	120	244.7–113.8	4	4
Arg-, and glu- cell	180	147.2–167.9	5	34
Serine-, cys., and alanine-MCNPs	50	116.5–85.3	4	7
Salicylideneimine/hydrothermal carbon	120	261.8	6	8
Amberltie IRA-910 resin	120	89.9	5	33
TEPA modified GMA-magnetic	60	409	4.5	42
TEPA- modified CS-magnetic	60	460.3	4	43
Phosphonate–mesoporous silica	30	303.3	6	44
PA-SBA-15	60	373.1	5.5	45
NH_3_-GO	120	80.13	6	40
M-IDA-PGMA	90	122.9	4	This study
M-IDP-PGMA	120	147.0	4	This study

Thermodynamics characteristics of uranium sorption at different temperatures were evaluated, based on the distribution coefficients *K*_d_ (L g^−1^): the ratio of *q*_eq_/*C*_eq_, for each temperature, *e.g.* enthalpy change (Δ*H*°), entropy change (Δ*S*°) by the van't Hoff equation (ln *K*_d_ = (−Δ*H*°/*R*)1/*T* + Δ*S*°/*R*), while the free energy (Δ*G*°) change can be deduced from (Δ*G*° = Δ*H*° − *T*Δ*S*°).^20,33^

**Fig. 9:**
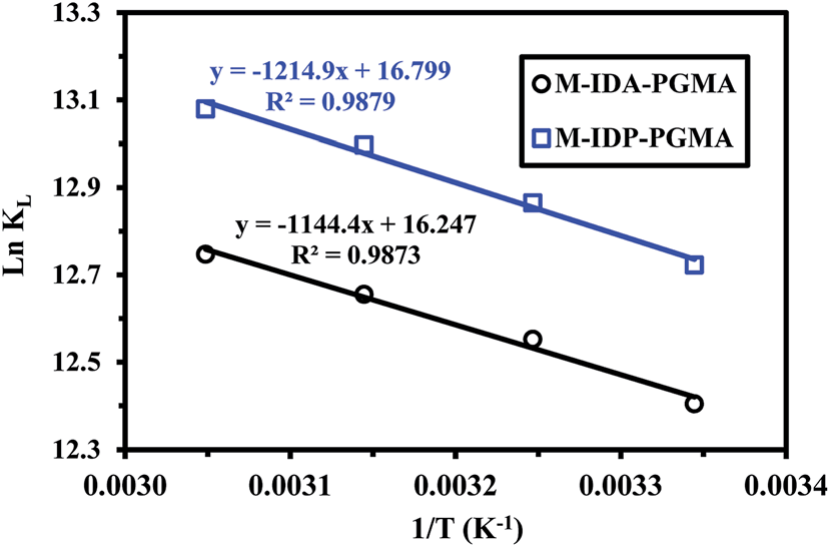
Thermodynamics of U(vi) sorption using M-IDA-PGMA and M-IDP-PGMA – van't Hoff plots of ln *K*_D_*vs.* 1/*T* (pH: 4.03; *C*_o_: 150 mg U L^−1^; time: 3 h; SD: 0.5 g L^−1^).

Fig. SI(9)† shows the very similar trends obtained with these materials for which sorption capacity increases with temperature. Fig. 9 shows the linear plots of ln *K*_d_*vs.* 1/*T*, and allows determination of the thermodynamic parameters of the different systems (Table 5) follow the same trends: (a) positive value of Δ*H*° (very close for the both sorbents: ranging between 9.5 and 10.1 kJ mol^−1^), indicating the endothermic nature of the sorption process. The global enthalpy changes consist of the combination of the dehydration enthalpy (Δ*H*_dehydr_, which is supposed to be positive due to the energy required for breaking the ion–water and water–water bonding of the hydrated metal ions) and the complexation enthalpy (Δ*H*_complex_, also positive),^6^ (b) positive value of Δ*S*° (with values very close), indicating an increase in randomness after metal sorption (at the solid/liquid interface) and this may be due to the release of water molecules bound to metal ions or the exchange of metal ions with more mobile ions (initially present on the sorbent).^6,35^ (c) negative value of Δ*G*° (the changes were of the same order of magnitude for both sorbents), indicating that the reaction is spontaneous (absolute value increases with temperature), and (d) the reaction is controlled by entropic changes than by enthalpy changes (|Δ*H*°| < |*T*Δ*S*°|).

**Table:** Thermodynamic parameters of U(vi) sorption

Sorbent	Temp. (K)	Δ*H*° (kJ mol^−1^)	Δ*S*° (J mol^−1^)	Δ*G*° (kJ mol^−1^)	*T*Δ*S*° (kJ mol^−1^)	*R* ^2^
M-IDA-PGMA	299	9.51	135.08	−28.47	40.39	0.987
	308			−29.50	41.60	
	318			−30.65	42.96	
	328			−31.80	44.31	
M-IDP-PGMA	299	10.10	139.67	−25.74	41.76	0.988
	308			−26.68	43.02	
	318			−27.73	44.42	
	328			−28.79	45.81	

#### 3.2.5. Metal desorption and sorbent recycling

Metal desorption is a key step in the design of a sorption system, indeed, this is the best way to improve the concentration of the target metal for final recovery or elimination. But this is also important for testing the recyclability of the sorbent which is critical for the economic competitiveness of the global process. Preliminary tests showed which a solution of sodium bicarbonate solution (0.25 M) efficiently desorbed uranyl ions: 90 minutes were sufficient for achieving efficient desorption.^4^ Using bicarbonate (NaHCO_3_) solutions avoids using acidic solutions that could damage the magnetite compartment of the sorbent. The ability of carbonate to form complexes with uranyl species (such as UO_2_(CO_3_)_2_^2−^, UO_2_(CO_3_)_3_^4−^, *etc.*)^4,46,47^ may explain the efficient elution of uranyl ions. Kabay *et al.*^46^ considered the possibility to reduce U(vi) to U(iv) for improving the desorption from a series of functionalized resins; actually, Na_2_CO_3_ or NH_4_CO_3_ solutions were highly efficient for metal desorption. In the case of magnetic chitosan particles loaded with uranyl ions, NaHCO_3_ solution was preferred against calcium oxalate for metal elution.^47^

Table 6 reports sorption and desorption steps repeated five times. The sorption and desorption efficiencies slightly decreases at each sorption stage: the loss in sorption capacity and efficiency with a limited loss in efficiency does not exceed 13% at the fifth sorption/desorption cycle. Sorbents have a good durability and stability in terms of sorption capacities. The possibility to easy separated, regenerated and recover the sorbents by external magnetic field contributes to make these materials very interesting for applications in hazardous conditions.

**Table:** Metal desorption and sorbent recycling – relative yields

Cycle no.	M-IDA-PGMA		M-IDP-PGMA	
	De (%)	Re (%)	De (%)	Re (%)
1	97.86	100^a^	96.93	100^a^
2	97.22	96.32	96.7	95.86
3	95.84	94.53	94.58	92.92
4	94.99	92.84	92.71	90.62
5	93.86	91.59	89.24	87.07

a Reference value for metal ion sorption efficiency (at first cycle) (NaHCO_3_: 0.25 M; *T*: 26 °C; sorbent dose: 2.0 g L^−1^; time: 1.5 h).

#### 3.2.6. Testing on sulfuric-acid uranium leachates of the El-Sella mining area

Acidic agitation leaching for selective uranium leaching, was operated under the following conditions: ore grinding with particle size below 100 mesh, H_2_SO_4_ concentration: 50 g L^−1^, temperature: 50 °C, solid/liquid ratio: 1 : 3, and contact time: 6 h.^4^ After the end of the leaching experiment, washing the solid ore residue with distilled hot water and finally filtered off. The obtained filtrate and washes were adjusted to certain volume representing the leach liquor which was analyzed for its U contents. The acid leachate pH was about 2.11; which was raised to 3.5–4.0 to cause iron precipitation and partial loss of uranium (about 91 mg L^−1^). The residual uranium concentration was 885.31 mg L^−1^ in the pregnant leaching liquor. Quantitative uranium sorption experiment from this sulfate liquor was achieved at initial pH 4.0, sorbent dose: 0.5 g L^−1^, in 180 min, and at room temperature 26 °C. Experimental results showed that the uranium content in the solutions after sorption were reduced from 885.31 mg L^−1^ to 834.21 and 845.68 mg L^−1^ for M-IDP- and M-IDA-PGMA respectively, showing that the sorption capacities were 102.2 and 73.3 mg U g^−1^, respectively. This about 15.7% and 26.5% lower than the maximum sorption capacities obtained in synthetic pure solutions. The high different concentrations and complicated aqueous acidic liquor composition and foreign rival ions, make the selectivity test sorption so difficult. Meanwhile, the sorbents preserve their high uranium sorption efficiency, as well as, M-IDP-PGMA is more selective for U(vi) than M-IDA-PGMA sorbent.

## 4. Conclusion

Two core–shell multifunctional magnetic-nanocomposites have been prepared suitably to be used as sorbents using facile two steps. Polyglycidylmethacrylate micro-particles (PGMA) was first functionalized with amino and finally by *N*-methylphosphonation, and *N*-methylcarboxylation prior to be incorporated to magnetite nanoparticles in a planetary ball milling apparatus to form magnetic core–shell sorbents (M-IDP-PGMA), and (M-IDA-PGMA), respectively. These nanocomposites were characterized by a series of techniques like elemental analysis, FTIR, XRD, pH_ZPC_, TEM, and VSM.

Sorbents are efficient for uranyl sorption at pH close to 4–5. Uptake kinetics were efficiently modeled using the PSORE: though most of the sorption occurs within 90–120 min. Sorption isotherms were fitted by Langmuir equation for which maximum sorption capacities were found to be 99.6 and 121.2 mg U g^−1^ for M-IDA- and M-IDP-PGMA sorbents, respectively (at *C*_o_: 150 mg L^−1^ and temperature 26 ± 1 °C). The sorption process was endothermic and spontaneous with increasing the randomness of the system. Conclusions of uptake kinetics, sorption isotherms and adsorption activation energy suggested that a dual mechanism of physic- and chemisorption owing to heterogenous binding sites.

Uranium can be efficiently regenerated from loaded sorbents using 0.25 M solutions of NaHCO_3_ and the sorbents can be re-used for at least 5/6 sorption/desorption cycles with a limited loss of sorption capacity (12%). Finally, these sorbents were successfully tested for uranium recovery from acidic sulfate liquor produced by sulfuric acid treatment of Egyptian ore from El-Sella area showing that despite its complex composition the sorbents maintained relatively high sorption capacities (maximum sorption capacities being reduced by less than 27% and 16% for M-IDA- and M-IDP-PGMA, respectively). The magnetic properties allowed the use of the material in hazardous environment with enhanced mass transfer characteristics with high sorption capacities.

## Conflicts of interest

There are no conflicts to declare.

## Supplementary Material

RA-009-C9RA06874K-s001
