# Supplementary material for: Facile synthesis of functionalized polyglycidyl methacrylate-magnetic nanocomposites for enhanced uranium sorption
Source: RSC Adv. 2019 Nov 27;9(66):38783–96. doi: 10.1039/c9ra06874k (PMC9075951; doi:10.1039/c9ra06874k)
Supplement: RA-009-C9RA06874K-s001 [file RA-009-C9RA06874K-s001.pdf]

Electronic Supplementary Material (ESI) for RSC Advances.
This journal is © The Royal Society of Chemistry 2019

Facile synthesis of functionalized polyglycidyl methacrylate-magnetic nanocomposites for enhanced uranium sorption

Ahmed A. Galhoum

Nuclear Materials Authority, P.O. Box 530, El-Maadi, Cairo, Egypt.

Supplementary Information

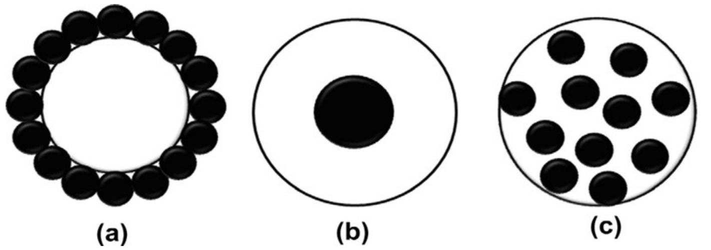

Figure SI1: Schematic representation of different structures of magnetic polymer beads **[16]**:

a) The Magnetic polymer beads are prepared from preformed polymer and magnetite particles by co-precipitation of iron salts directly in polymer matrix. In this case, polymeric matrix limits the growth of magnetic particles. Where the iron salts do not penetrate inside the beads and co-precipitate on their surface (Fig. S1a).

b) When magnetic particles are prepared by encapsulation of magnetic particles into block-copolymer micelles, which gives magnetic core-polymer shell type structures (Fig. S1b).

c) When the magnetic particles are homogeneously dispersed within the structure of polymer matrix (Fig. S1c).

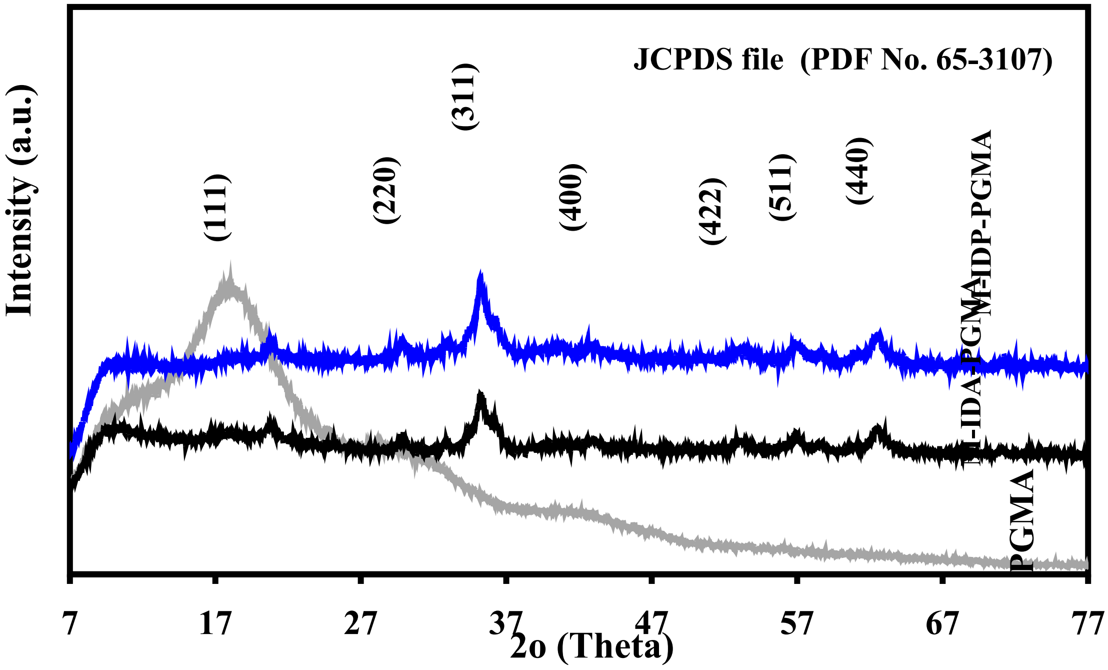

Figure SI2. XRD analysis of the PGMA, M-IDA-, and M-IDP-PGMA IDP-PGMA sorbents.

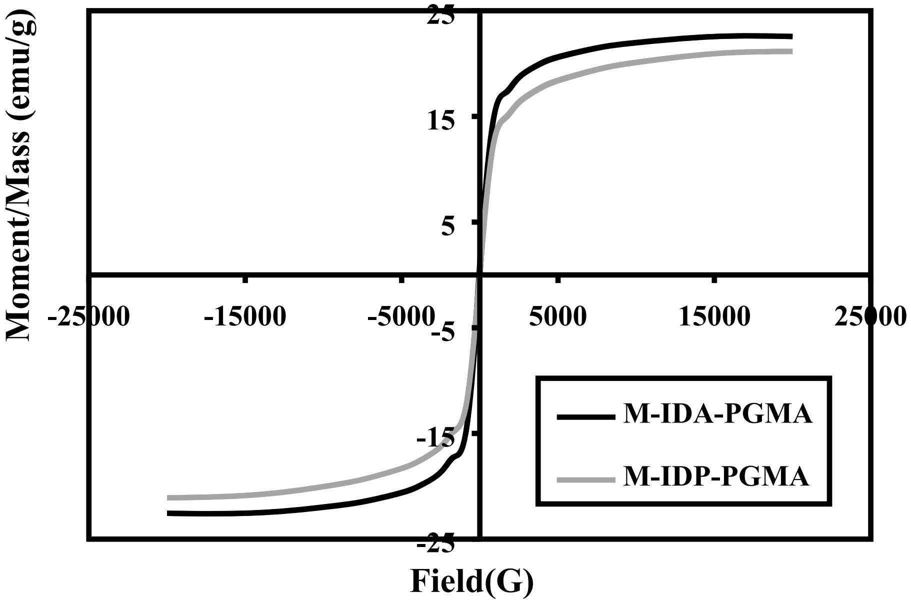

Figure SI3. Magnetization curves of M-IDA- and M-IDP-PGMA sorbents.

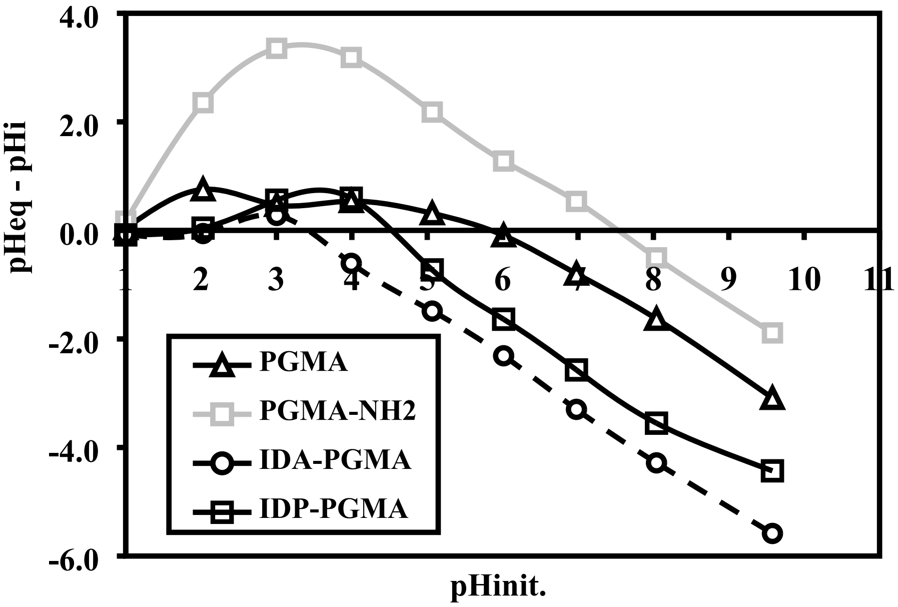

Figure SI4: Determination of pH_{ZPC} for the IDA-PGMA and IDP-PGMA sorbents. (SD: 2 g L^{-1}; Background salt: NaCl (0.1 M); agitation time: 24 h).

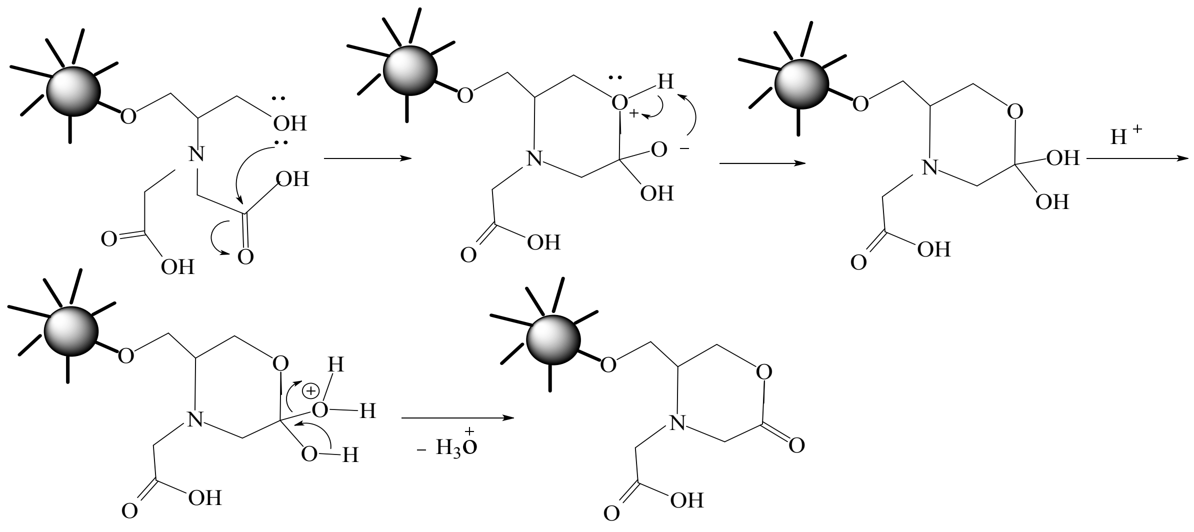

Figure SI5: Revised process for the lactone sixth membered ring of IDA-PGMA.

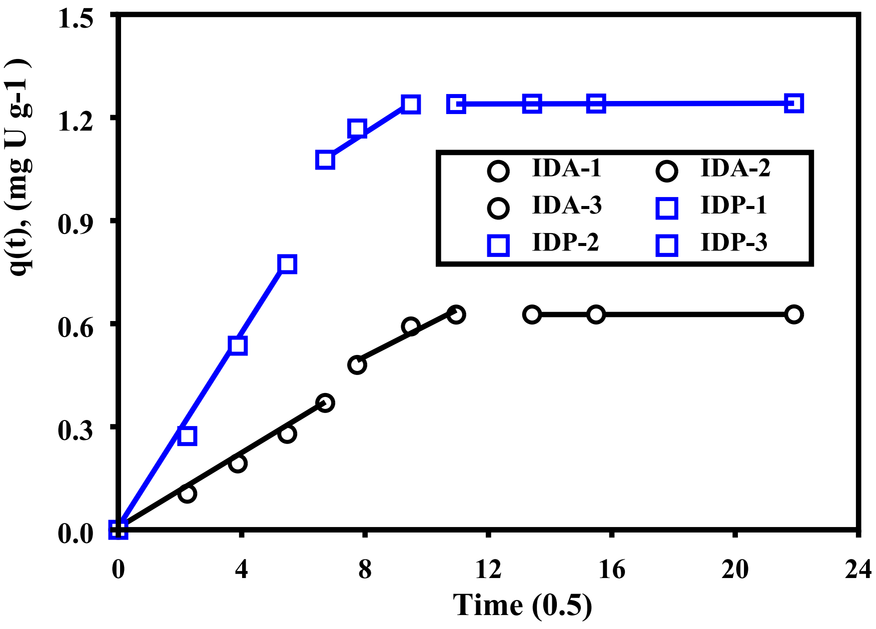

Figure SI6: kinetic profiles with the simplified equation of resistance to intraparticle diffusion (sRIDE, Weber and Morris equation) Multi-linear plots of the sRIDE: q(t) vs. $t^{(0.5)}$ (pH_o: 4.02; C_o: 100 mg U ion L^{-1}; T: 26 ^oC; SD: 0.5 mg L^{-1}).

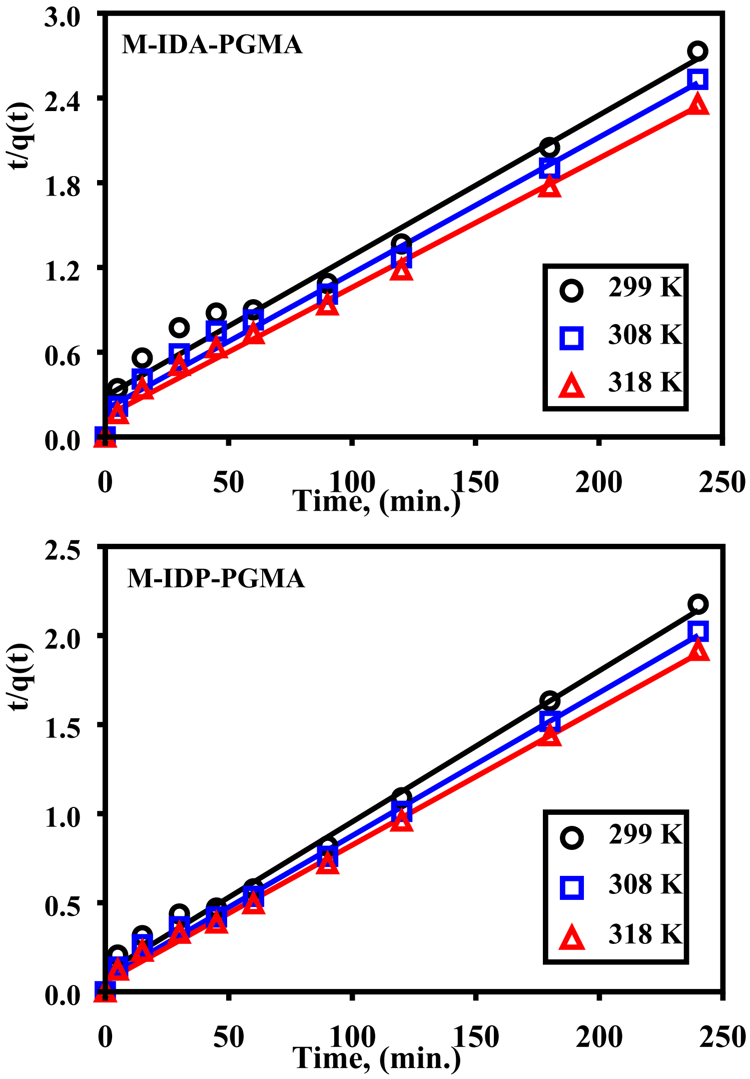

Figure SI7: kinetic profiles for the linearization of PSORE for U(VI) sorption. (pH_o: 4;0; C_o: 100 mg U L^{-1}; SD: 0.5 mg L^{-1}).

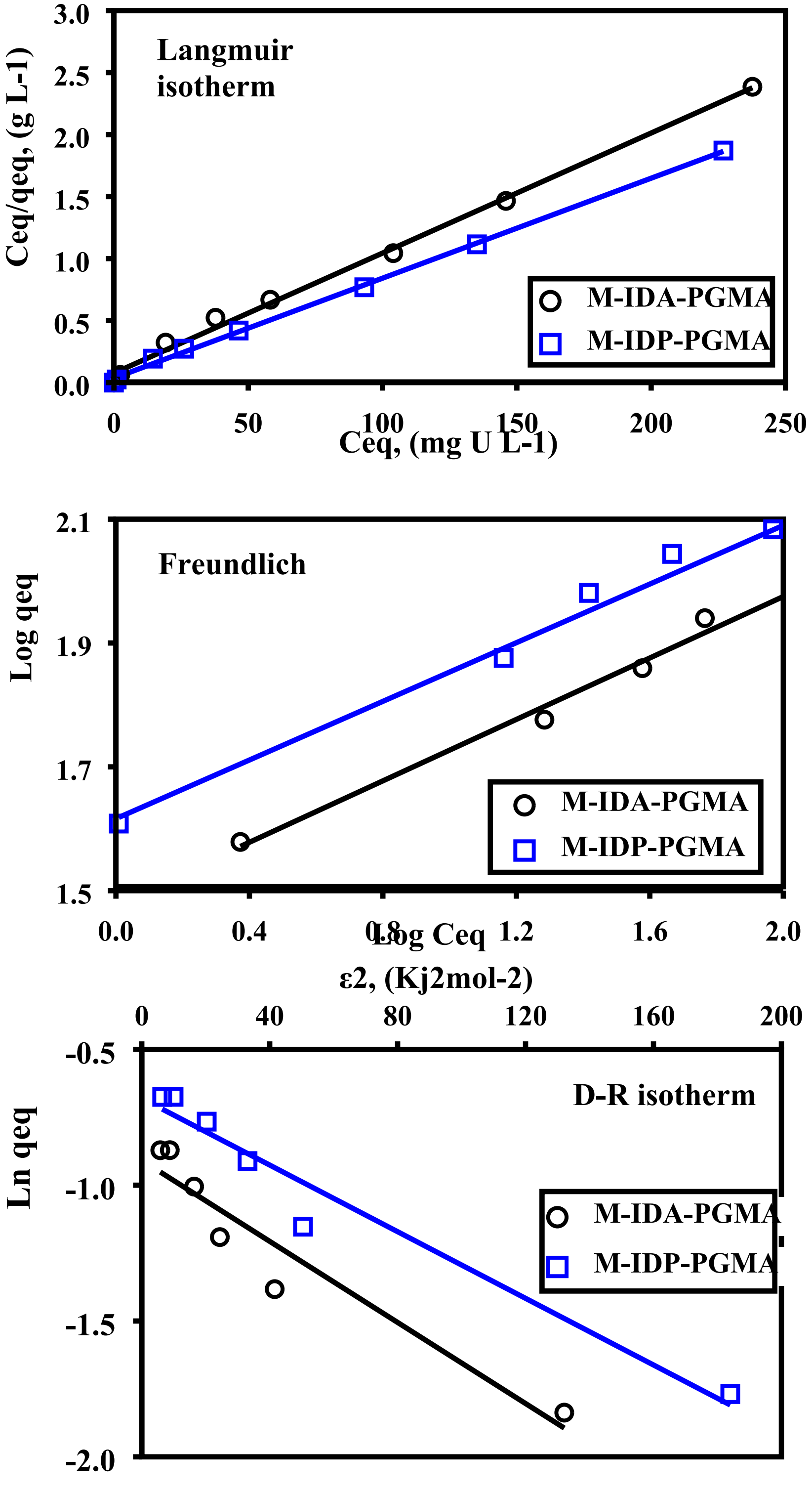

Figure SI8: Linearization of Langmuir, Freundlich, and D-R model plots for U(VI) sorption. (pH_o: 4.0; T: 26 ^{o}C; SD: 0.5 g L^{-1}; Time: 12 h).

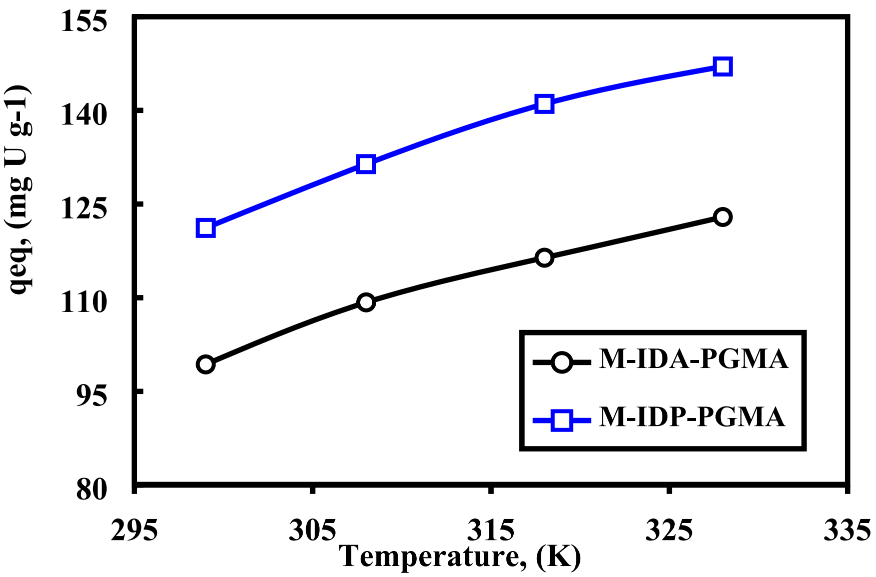

Figure SI9: Thermodynamic study of U(VI) sorption: variation of q_{max} with temperature (pH_o: 4.0; C_o: 150 mg U L^{-1}; Time: 2 h; SD: 0.5 g/L).

Table SI1: Sorption modeling: kinetics and sorption isotherms.[35]

Process	Model	Equation	Parameters	
**Kinetics**	PSORE	$q(t) = \frac{q_{eq,2}^{2} \times k_2 \times t}{1 + q_{eq,2} \times k_2 \times t}$	$q_{eq,2}$ (mg U g^{-1})	k_2 (L mg^{-1} min^{-1})
	sRIDE (Weber & Morris)	$q_{(t)} = k_{int.,i}.\ t^{0.5} + C$<br><br>Several linear sections corresponding to different regimes of resistance (i) to intraparticle diffusion may co-exist ($K_{int,i}$) (linear regression calculation)	$K_{int.,i}$ (mg g^{-1} $min^{-0.5}$)	
**Isotherms**	Langmuir	$\frac{C_{eq}}{q_{eq}} = \frac{C_{eq}}{q_{max}} + \frac{1}{bq_{max}}$	q_{max} (mg U g^{-1})	b_L (L mg^{-1})
	Freundlich	$q = k_F\, C_{eq}^{1/n}$	k_F	n (dimensionless)
	D-R (Dubinnin-Radushkevich)	$lnq_{eq} = lnq_{m,S} - k\varepsilon^2$<br>$E_{DR} = (2k)^{-1/2}$	$q_{m,S}$	E_{DR}

Section II - Issues on reproducibility of synthesis and sorption properties

In order to evaluate the reproducibility in the synthesis of the sorbents two batches of ID-PGMA and IDP-PGMA were produced. Table below compares the CHN element analysis (weight %).

Sample	Batch	C (%)	H (%)	N (%)	P (%)
PGMA	# 1	57.78<br>57.81	7.27<br>7.24	0.33<br>0.28	---<br>---
PGMA-NH_2	# 1	49.64<br>49.72	7.46<br>7.32	3.57<br>3.61	---<br>---
IDA-PGMA	# 1	43.02<br>42.92	7.57<br>7.44	2.23<br>2.24	---<br>---
IDP-PGMA	# 1	40.78<br>40.94	6.90<br>6.95	2.01<br>1.92	1.78<br>1.72
M-IDA-GMA	# 1	28.49<br>28.52	4.76<br>4.69	1.58<br>1.49	---<br>---
M-IDP-GMA	# 1	26.97<br>26.90	4.46<br>4.41	1.27<br>1.19	1.19<br>1.11
PGMA	# 2	57.01<br>56.81	7.15<br>7.33	0.18<br>0.25	---<br>---
PGMA-NH2	# 2	48.92<br>48.86	7.28<br>7.02	3.31<br>3.31	---<br>---
IDA-PGMA	# 2	42.24<br>42.08	7.42<br>7.49	2.11<br>2.08	---<br>---
IDP-PGMA	# 2	39.97<br>39.91	6.81<br>6.77	1.98<br>1.93	1.92<br>1.83

The two adsorption experiments were compared for selected experimental conditions in terms of pH effect, uptake kinetics, sorption isotherms and temperature effect (under comparable conditions than those reported in the core manuscript.

For nano-structure characterization – Transmission electron microscopy and crystalline structure – X-ray diffraction pattern: the ball-milling process with different types of functionalized PGMA was repeated, under the same selected experimental conditions and the XRD analysis and TEM analysis for these materials give the same results (**Not published for the moment, yet, in comment part**). This confirms the idea of the new route for manufacturing of magnetic nanocomposites using a facile solid-state method: by milling together pre-formed magnetic nanoparticles and functionalized PGMA, was confirmed. **N.B.:** I did not repeat the ball-milling process for the same functionalized PGMA (IDA-PGMA, and IDP-PGMA) nanocomposite core-shell.
